# Determination of Prostaglandins (Carboprost, Cloprostenol, Dinoprost, Dinoprostone, Misoprostol, Sulprostone) by UHPLC-MS/MS in Toxicological Investigations

**DOI:** 10.3390/toxics11100802

**Published:** 2023-09-22

**Authors:** Paweł Szpot, Olga Wachełko, Marcin Zawadzki

**Affiliations:** 1 Department of Forensic Medicine, Wroclaw Medical University, 4 J. Mikulicza-Radeckiego Street, 50345 Wroclaw, Poland; 2 Institute of Toxicology Research, 45 Kasztanowa Street, 55093 Borowa, Poland; 3 Faculty of Medicine, Wroclaw University of Science and Technology, Wybrzeże Wyspiańskiego 27, 50370 Wroclaw, Poland

**Keywords:** prostaglandins, UHPLC-QqQ-MS/MS, pharmaceutical drugs, abortifacients, toxicological analysis

## Abstract

Prostaglandins have stimulative influence on the human uterus and therefore were introduced to medical treatment in reproductive healthcare as labor inductors or abortifacients. The UHPLC-ESI-QqQ-MS/MS method was developed for six prostaglandins: carboprost, cloprostenol, dinoprost (PGF2α), dinoprostone (PGE2), misoprostol and sulprostone (substances for pregnancy termination) in pharmaceutical samples and was applied for the toxicological examination of pills containing misoprostol (collected during gynecological examination). There were used two internal standards: misoprostol-*d_5_* and PGF2α-*d_4_*. The quantification of analytes was performed in the MRM mode. The linearity of method was in the range from 0.1 to 10 µg/mL, with a coefficient of determination above 0.997 (*R*^2^) for each compound. The precision and accuracy values did not exceed ±5.0%. Analysis of the pills revealed the presence of two substances: misoprostol and diclofenac. Misoprostol and diclofenac dose per sample were as follows: 608.8 ng (sample 1), 708.4 ng (sample 2), 618.8 ng (sample 3) and 67.7 mg (sample 1), 65.3 mg (sample 2) 67.3 mg (sample 3), respectively. A simple, precise and reliable method can be applied for routine examinations in terms of clinical and forensic toxicology examinations as well as in quality control of drugs for pharmaceutical purposes (original drugs and counterfeit medications).

## 1. Introduction

**Figure 1:**
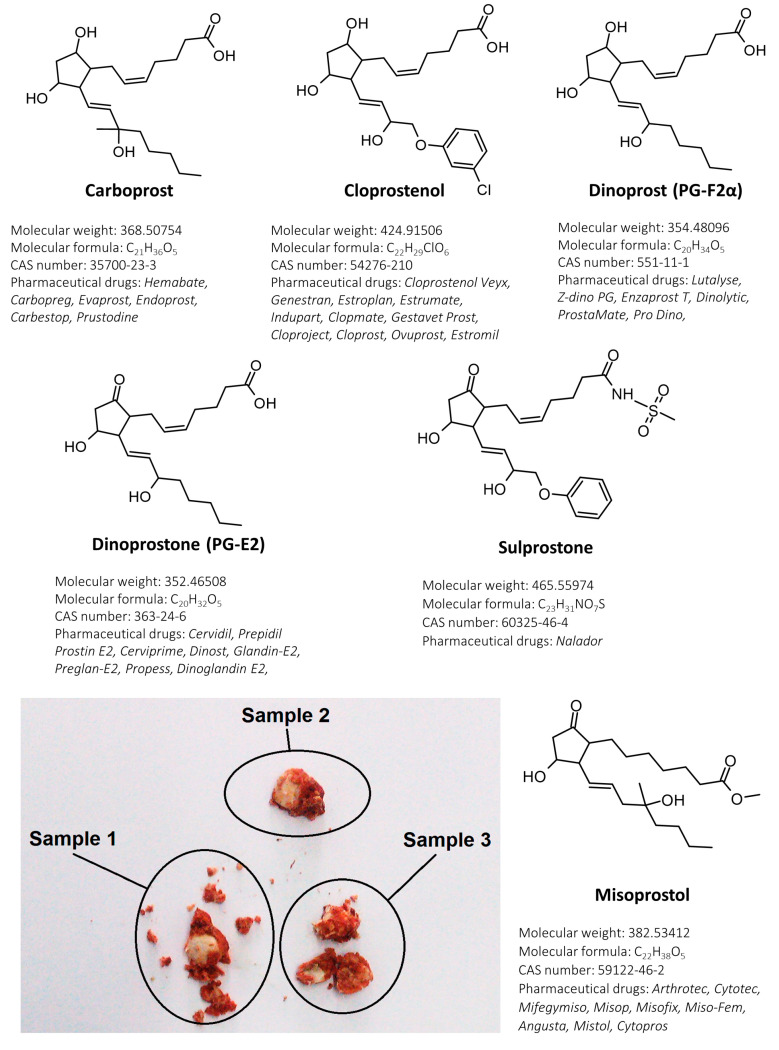
Chemical structures of determined compounds (with short description of each prostaglandin) and pills collected during gynecological examination with visible fragments of myometrium and blot clots.

Prostaglandins are physiologically active lipid autacoids that are formed during arachidonic acid transformations. Some of these compounds have a potent, stimulative influence on the human uterus and therefore were introduced to medical treatment in reproductive healthcare as labor inductors or abortifacients. To date, some of primary prostaglandins: alprostadil (PGE1), dinoprostone (PGE2) and dinoprost (PGF2α); or their synthetic analogues (misoprostol, carboprost, cloprostenol, sulprostone) have been used worldwide for pregnancy termination [1]. Data collected between 2015 and 2019 revealed that there were more than 120 mln unintentional pregnancies per year and 61% of this gestations ended in an abortion [2]. Pharmaceutical abortion is a safe method with a success rate of 97%, however, when performed at home without medical supervision or specialistic care, it can pose a great public health problem. Taking into consideration the years between 2010 and 2014, almost half of analyzed abortions each year were unsafe. The problem with unsafe pregnancy terminations is much higher in developing countries (49.5%) than in developed ones (12.5%) [3]. A retrospective study conducted by Nivedita et al. [4] on self-induced abortion cases showed that 62.5% patients were found to have incomplete abortion and 7.5% ended in sepsis. Furthermore, out of 128 women, 40 patients obtained pills from the internet without medical consultation. An analysis of 22 products bought on 20 websites performed by Murtagh et al. [5] revealed that in the majority of pills with misoprostol, this substance was in a lower concentration than declared on the label. In some cases [6], illegally distributed medications (advertised by the vendor as “abortion pills”) contain only lactose (without any active ingredient). Counterfeit drugs are distributed on illegal online marketplaces without any legislation and pharmaceutical control. Out of 109 abortion-related websites analyzed by Kerestes et al. [7], 22.6% sold medications for pregnancy termination. It is worth noticing the fact that pills of an unknown origin (bought on the Internet) may not contain the appropriate concentration of the active ingredient and/or may include hazardous and toxic impurities. The original pharmaceutical products (e.g., Hemabate^®^, Cervidil^®^, Arthrotec^®^, Cytotec^®^) containing prostaglandins are summarized in Figure 1.

To date, the methods for prostaglandins determination were mainly concerned on analysis of biological samples, e.g., human menstrual fluid (PGE2 and PGF2α) [8]; saliva (PGE2) [9]; monkey plasma (sulprostone) [10]; dog plasma (carboprost) [11]; or human whole blood, placenta and fetal liver (misoprostol acid) [12]. However, approaches and methodologies for pharmaceutical analysis are still a scarcely explored topic. It was pointed out in the 90s that access to healthcare information from unsupervised sources through the Internet would increase the potentially unsafe use of counterfeit misoprostol in self-induced abortions [13]. Even today, there is still a lack of sufficient data to determine the safety of abortion pills sourced online, therefore further investigations are needed. Only a few methods such as FTIR [14], spectrophotometry [15], HPLC with UV detection [16] and tandem mass spectrometry [17,18] were applied for pharmaceutical drugs analysis.

This paper aims to develop a simple, sensitive and precise UHPLC-QqQ-MS/MS method for the determination of six prostaglandins used in pharmacological pregnancy termination. This technique was fully validated and applied in an authentic toxicological case of abortion pills analysis.

In toxicological examinations, blood and urine samples are collected most often. However, it is worth noticing alternative materials, i.e., oral fluid, hair, meconium, breast milk [19], vitreous humor, bone marrow [20], exhumated samples (e.g., bones) [21] and entomological specimens [22]. In this paper’s authentic casework, we present a toxicological analysis of pills collected during gynecological examination.

## 2. Materials and Methods

### 2.1. Chemicals and Reagents

Water and methanol (Chromasolv^®^ LC-MS; Witko, Łódź, Poland) and formic acid were purchased from Sigma–Aldrich (Steinheim, Germany); ammonium formate was purchased from Sigma–Aldrich (Mumbai, India); carboprost, cloprostenol, misoprostol, PGE2, PGF2α and misoprostol-*d_5_* were purchased from TRC (Toronto, ON, Canada); sulprostone was purchased from Sigma–Aldrich (St.Louis, MO, USA); PGF2α-*d_4_* was purchased from ChemCruz (Santa Cruz Biotechnology Inc.; Dallas, TX, USA). Standards in the form of neat powders were dissolved in methanol. The stock standard solutions of determined substances were mixed to obtain a working solution at a concentration of 100 µg/mL. Deutered analogues were mixed to obtain a methanolic solution at a concentration of 1 µg/mL. The standard solutions were stored at a temperature of −20 °C. Drug-free blank blood samples, used for the examination of the applied method’s selectivity, were obtained from the Regional Blood Donation Center.

### 2.2. Instrumentation

Analyses were performed using a UHPLC (Shimadzu Nexera LC-40D XS; Kyoto, Japan). Separation was performed using an Acquity UPLC BEH C18 column (50 × 2.1 mm i.d., particle size 1.7 µm; Waters, Milford, MA, USA) with the thermostat set at 40 °C. The mobile phase consisted of (A) water with 10mM HCOONH4 and 0.1% HCOOH, and (B) methanol with 10mM HCOONH4 and 0.1% HCOOH. The gradient elution was carried out at a constant flow of 0.3 mL/min. The gradient applied was as follows: 0 min, 5% B; 6.0 min, 95% B; 8.0 min, 95% B, and 8.1 min 5% B. Return to the initial gradient compositions (95% A/5% B) was performed for 5 min.

Detection of the investigated compounds was achieved using a triple-quadrupole mass spectrometer (QqQ, Shimadzu 8060, Kyoto, Japan). The spectrometer was equipped with an ESI source; determination of the investigated substances was carried out in the MRM mode. The following MS parameters were fixed: nebulizing gas flow, 3 L/min; heating gas flow, 10 L/min; interface temperature, 300 °C; desolvation line temperature, 250 °C; heat block temperature, 400 °C; and drying gas flow, 10 L/min. A summary of precursor and product ions, collision energies, dwell time, Q1–Q3 pre-bias voltages, and retention time for each compound is presented in Table 1.

### 2.3. Working Solutions, Calibration Curve and Sample Preparation

The working standard solution was diluted with methanol to achieve the following concentrations of prostaglandins: 0.1, 0.25, 0.5, 1, 2.5, 5 and 10 µg/mL. Next, 10 µL of each calibration level was transferred to 2mL-Eppendorf tube, mixed with 80 µL methanol and 10 µL of IS solution (mix of misoprostol-*d_5_* and PGF2α-*d_4_*; each substance at concentration of 1 µg/mL). Tubes were centrifuged at 13,500 rpm for 1 min and transferred to glass inserts for UHPLC-QqQ-MS/MS analysis. Injection volume was 2.0 µL. Quality control samples (QC) used in validation were in concentrations of 0.1 (low QC), 1 (medium QC) and 10 (high QC) µg/mL. Authentic forensic samples (pills collected during gynecological examination) were divided into three parts (and signed as sample 1, sample 2, sample 3) and each sample was transferred to a separate 12-mL plastic tube. Next, 5 mL of ultra-pure methanol was added and samples were ultrasonicated by 15 min. Eventually, the mixture was centrifuged at 4,000 rpm for 5 min and 10 µL of sample was mixed with 80 µL of methanol and 10 µL of IS solution.

### 2.4. Validation

Validation of the method included the examination of linearity, precision and accuracy, carryover, LOD and LOQ, stability of prostaglandins and selectivity of the method. All validation parameters (except selectivity) were evaluated using methanol working solutions at three concentrations of 0.1, 1 and 10 µg/mL. Linearity was evaluated by examination of prostaglandins’ working solutions in the calibration range of 0.1–10 µg/mL. Linear calibration model was applied. The coefficient of determination (*R*^2^) was determined. Precision and accuracy were estimated by replicating the analysis (n = 3) of samples at three concentration levels: 0.1, 1 and 10 µg/mL. Precision was defined as RSD%, and accuracy was expressed as RE%. Intra-day precision and accuracy were evaluated by analyzing QC samples over 1 day, while inter-day values were evaluated by analyzing QC samples on three different days. To investigate the carryover, three samples with internal standards (without analytes) only were analyzed after a calibration sample at the highest calibration level (10 µg/mL). Unacceptable carryover was considered to be when a peak area ratio in a zero sample would exceed 20% of the area ratio observed for the LOQ samples. The LOQ was defined as the concentration at which the RSD% does not exceed 20% and the signal-to-noise ratio met the condition at least: S/N ≥ 10. Due to the fact that pills analyzed in this paper contained blood clots on their surface (a complex biological matrix that contains many different low-molecular-weight chemical compounds that can affect the determination), selectivity was tested by analyzing the blank blood extract. The sample was prepared as follows: 100 µL of blood was precipitated with 200 µL of ice-cold methanol, centrifuged, and 10 µL of methanolic extract was transferred to the glass insert. Next, 80 µL of methanol and 10 µL of IS MIX solution were added. Stability of prostaglandins were examined for three concentration levels (0.1, 1 and 10 µg/mL) by analyzing the sample immediately after preparation and later after 1.5, 3, 4.5, 6, 7.5, 9, 10.5, 12, 26 and 45 h. Samples were stored in an autosampler at a stable temperature of 5 °C.

## 3. Results

### 3.1. Optimization of Mass Spectrometry Parameters

**Figure 2:**
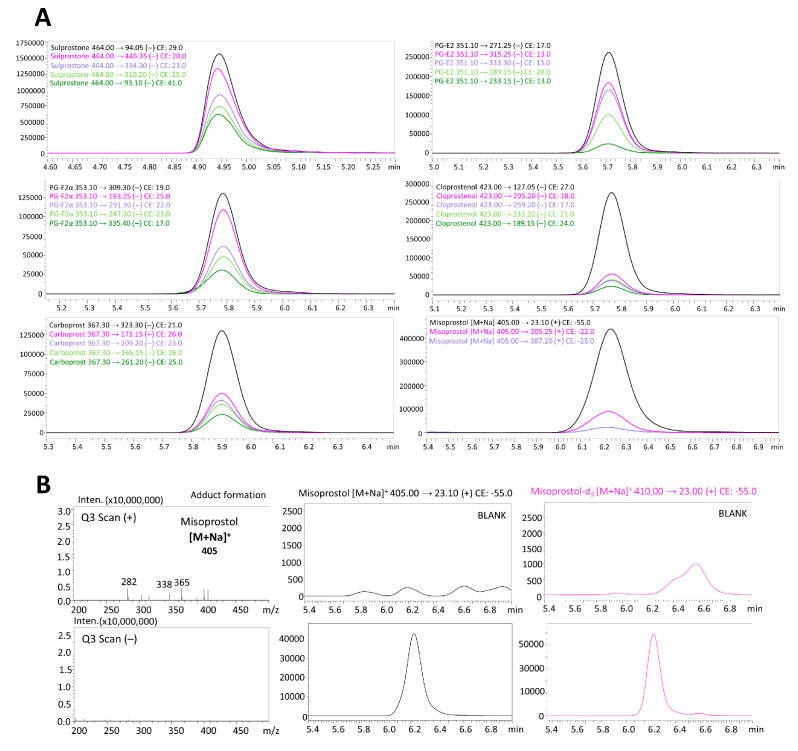
(**A**) Multiple reaction monitoring (MRM) chromatograms of determined prostaglandins; and (**B**) misoprostol adduct formation in Q3 (+/−) scan mass spectra with quantitative MRM transition of misoprostol and misoprostol-*d_5_*.

**Figure 3:**
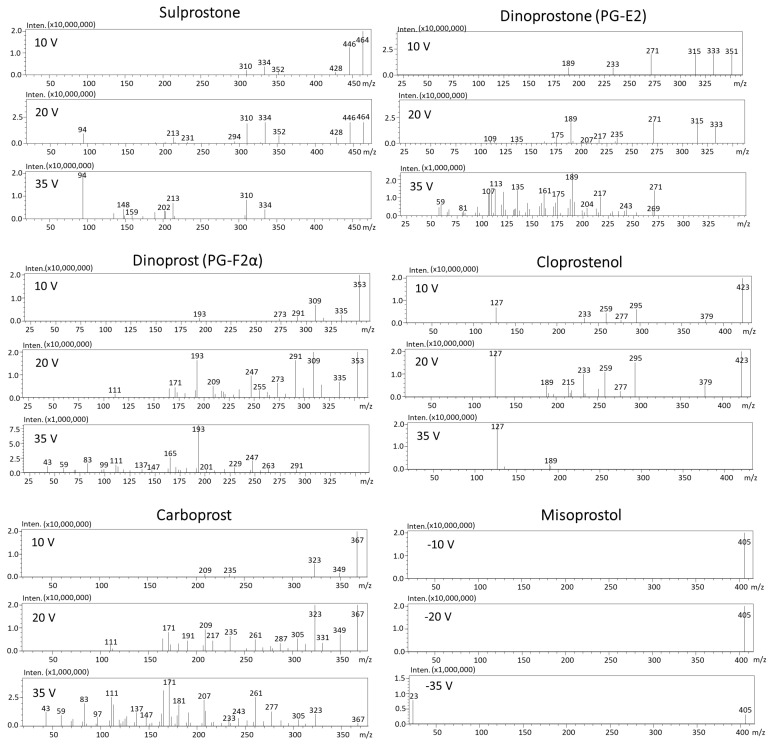
Product ion scan mass spectra of sulprostone, dinoprostone, dinoprost, cloprostenol, carboprost (in negative ionization mode) as well as misoprostol (in positive ionization mode).

In order to select the best ionization mode, a Q3 scan (+ and −) in the mass range of 50–500 *m*/*z* was performed before automatized optimization with the use of MRM optimization software. The standard solution of each substance at concentration of 1 µg/mL was subjected directly to mass spectrometry (without chromatographic column). Precursor ions of all prostaglandins were observed in negative ionization mode [M−H]^−^, except for misoprostol and misoprstol-*d_5_*, which formed sodium adducts in positive ionization mode [M+Na]^+^ (Figure 2B). Adducts in positive ionization mode can by effectively used as precursor ions [23]. The possibility of misoprostol’s adduct formation in ESI was previously observed by Chu et al. [18]. In order to collect product ion scan mass spectra, each substance at a concentration of 10 µg/mL was subjected to chromatographic column. The results of precursor fragmentation are presented in Figure 3. For all determined compounds, five MRM transitions were selected (product ions with the highest intensities), except for misoprostol and misoprostol-*d_5_*, which provided only three product ions (the most intensive one was 23 *m*/*z* which corresponds to sodium Na^+^). The MRM chromatograms of determined prostaglandins are presented in Figure 2A.

### 3.2. Validation Results

The LOQ was 0.1 µg/mL for all determined compounds. The linear concentration range was from 0.1 to 10 µg/mL. The coefficients of determination (*R*^2^) were above 0.997 for all substances. The precision and accuracy values did not exceed ±5.0%. Furthermore, there were no carryover between the samples. The results of the validation process are presented in Table 2. The stability study revealed that all substances were stable for up to 45 h. Fluctuations in the concentrations were not greater than the accuracy of the method. During selectivity studies, there was no signal enhancement by any ions from the biological matrix, except 247 *m*/*z* for PGF2a (intensity below 250) and 271 *m*/*z* for PGE2 (however, at a retention time that was shifted by 0.15 min relative to the PGE2-specific RT).

### 3.3. Method Application and Toxicological Findings

A 26-year-old female patient (pregnancy 3, miscarriage 3) was transported to the hospital after preterm labor at home. The gynecological examination revealed no external or internal lesions (uterus contracted in normal dimensions, moderate bleeding). Examination with the use of a vaginal speculum found vaginal laxity with placental fragments. In posterior vaginal fornix there were blood clots with three white pills (Figure 1). All pills were collected for toxicological examination. The patient claimed that pills contained antifungal substances. Toxicological analysis of the pills (collected during gynecological examination from a vagina) revealed the presence of two substances: misoprostol and diclofenac. Misoprostol dose per sample were as follows: 608.8 ng (sample 1), 708.4 ng (sample 2) and 618.8 ng (sample 3). Diclofenac dose per sample were as follows: 67.7 mg (sample 1), 65.3 mg (sample 2) and 67.3 mg (sample 3). Diclofenac was quantified with the use of the method published earlier [21]. No antifungal substances were found in the tested samples.

## 4. Discussion

Taking into consideration the presented results, we can hypothesize that the pills used to induce abortion could have been Arthrotec forte^®^ (containing 75 mg of diclofenac and 200 µg of misoprostol per pill), as this drug is the only one available in our country at the abovementioned doses of active ingredients. Concentrations of diclofenac and misoprostol in analyzed samples were much lower than declared in the package leaflet; however, it is worth noting the fact that these drugs were revealed from pills and part of them could have been absorbed in woman’s birth tract. Misoprostol may induce preterm labor, and the dose taken by the woman likely led to the termination of pregnancy [12].

The summarization of methods applied for the determination of prostaglandins used as abortifacients is presented in Table 3. These techniques were used in the analysis of original medications, injection liquids, infusion preparations, counterfeit drugs, as well as pills collected during gynecological examinations. Except for the methods developed by Focardi et al. [24] and Baskar et al. [14], other techniques enabled the quantitative analysis of the samples. The authors of abovementioned study [24] confirmed the use of misoprostol for self-induced abortion in the qualitative analysis by comparison of chromatograms obtained during the analysis of authentic samples with chromatograms obtained for Cytotec^®^ (pills used as reference material).

The majority of the described-to-date methods utilized chromatographic separation; however, in two papers, ATR-FTIR [14] and spectrophotometry [15] were used. Unfortunately, these methods are not applicable to the examination of medical products collected in authentic toxicological and clinical cases, e.g., pills collected from the vagina [24] or from food [25] (a case of a man who mixed abortifacients with meals to induce abortion in his pregnant girlfriend described by Watzer et al.). Also, the analysis of multicomponent medications of an unknown origin would be nearly impossible (especially in spectrophotometry) due to the overlapping of bands from different chemical compounds. Among chromatographic techniques, the most sensitive was that described by Lee et al. [17] with LOQ of 0.015 µg/mL. However, the upper limit of the quantification (0.3 µg/mL) of the method is definitely below the range of concentrations that could be found in the pharmaceutical drugs. For this reason, samples for testing must be diluted many times, which increases the bias of determination. The concentration range of the HPLC-UV technique developed by Vijayasree et al. [16] was the closest to the concentrations of carboprost in injection dosage form (ULOQ 249 µg/mL); however, this technique (similarly to that described above) would not be suitable for counterfeit drugs analysis and for quality control purposes. Such preparations may contain a variety of other substances that would be impossible to identify by UV detector without having reference standards. In addition, substances without chromophores will be invisible to the detector in this technique. Moreover, the methods presented in Table 3 were mainly dedicated to the analysis of limited number of substances (misoprostol and/or carboprost). Due to the fact that there is a significant number of various abortifacients containing prostaglandins as active ingredients, the methods should be developed for many different PGs. To our knowledge, the UHPLC-QqQ-MS/MS technique presented in this paper is the first quantitative method applied to date that allows simultaneous determination of the largest number of prostaglandins with abortifacient properties. In addition, by using two internal standards with different chemical structures: an analog of PGE1—misoprostol-*d_5_* (dedicated for analysis of PGs in positive ionization mode) and deuterated analog of PGF2α (dedicated for analysis in negative ionization mode), it is possible to perform a reliable, accurate and precise quantification of active drug components concentrations.

The securing of abortion pills for toxicological investigations is not a standard practice; however, such evidence can eventually be the only ones to confirm or exclude the use of abortifacients both by the mother [24,26] and by third parties for criminal purposes [25]. Examining the biological material is also a very important step in such cases, however, prostaglandins are rapidly absorbed and then metabolized [1,12,24], and are therefore very difficult to detect in biological samples in their initial form. For these reasons, metabolites of PGs, e.g., misoprostol acid (the active metabolite of misoprostol), can be determined; however, it is worth noting that this compound also exhibits relatively rapid metabolism and/or degradation [12]. Therefore, it may not be possible to detect this substance several days after its ingestion. The aforementioned analytical problems were encountered by Hopson et al. [26]. In the described case, pills from the vagina were collected during a gynecological examination, however, they were not sent for toxicological examinations. The analysis of placenta and fetal postmortem blood samples (performed several days after preterm labor) did not reveal the presence of abortifacients. This could have been caused by several factors, e.g., misoprostol acid had completely degraded or was metabolized by the fetus. Or misoprostol acid was present in fetal biological samples, however, at concentrations below the method’s detection range or the pills found in the woman’s vagina contained substances other than abortifacients (e.g., antifungals). Without toxicological analysis of the pills secured in this case, it is impossible to specify whether the cause of the fetal death was due to self-induced abortion with the use of misoprostol.

The method presented in this paper can be applied to the analysis of pills (as described in this paper) as well as other dosage forms of medical products (original as well as counterfeit drugs). Moreover, it has a potential to be applied as a routine analytical technique in clinical and forensic toxicology laboratories for the examination of evidence secured during prosecutions.

## 5. Conclusions

A sensitive and selective UHPLC-QqQ-MS/MS method for the determination of prostaglandins use in medical treatment as pharmaceutical drugs (carboprost, cloprostenol, dinoprost, dinoprostone, misoprostol, sulprostone) was developed and validated. The calibration range of the method was 0.1–10 ug/mL; precision and accuracy values did not exceed ±5.0%; coefficients of determination (*R*^2^) were >0.997 for all compounds. The method was applied in authentic forensic toxicological analysis of pills revealed during the gynecological examination of women suspected of performing self-induced pregnancy termination.

## Figures and Tables

**Table 1:** Multiple reaction monitoring (MRM) conditions used in the UHPLC/ESI-QqQ-MS/MS method for quantification of prostaglandins in pharmaceutical drugs.

No.	Substance	Ionization /Ion	Precursor Ion [*m*/*z*]	Product Ion [*m*/*z*]	Dwell Time (msec)	Q1 Pre-Bias [V]	Collision Energy [V]	Q3 Pre-Bias [V]	Retention Time [min]
1	Carboprost	Negative [M−H]^−^	367.3	323.3 *	1	19	21	12	5.914
				171.15	1	27	26	12	
				209.2	1	14	23	14	
				165.15	1	14	26	11	
				261.2	1	27	25	17	
2	Cloprostenol	Negative [M−H]^−^	423.0	127.05 *	1	13	27	23	5.775
				295.2	1	22	18	21	
				259.2	1	23	17	18	
				233.2	1	16	21	16	
				189.15	1	16	24	13	
3	Dinoprost (PGF2α)	Negative [M−H]^−^	353.1	309.3	1	26	19	11	5.791
				193.25 *	1	26	25	13	
				291.3	1	26	22	21	
				247.3	1	26	23	17	
				335.4	1	26	17	12	
4	Dinoprostone (PGE2)	Negative [M−H]^−^	351.1	271.25 *	1	18	17	13	5.719
				315.25	1	26	13	11	
				333.3	1	25	13	12	
				189.15	1	25	20	13	
				233.15	1	18	13	17	
5	Misoprostol	Positive [M+Na]^+^	405.0	23.1 *	1	−19	−55	−26	6.237
				305.25	1	−23	−22	−14	
				387.2	1	−25	−25	−13	
6	Sulprostone	Negative [M−H]^−^	464.0	446.35	1	11	20	16	4.951
				334.3	1	24	23	12	
				310.2	1	25	25	22	
				94.05 *	1	18	29	16	
				93.1	1	14	41	15	
IS1	Dinoprost-d4 (PGF2α-d4)	Negative [M−H]^−^	357.3	313.3 *	1	20	19	11	5.785
				197.25	1	19	25	14	
				295.4	1	19	22	21	
				251.3	1	19	23	16	
				213.3	1	19	17	15	
IS2	Misoprostol-d5	Positive [M+Na]^+^	410.0	23.0 *	1	−22	−55	−26	6.232
				305.2	1	−19	−22	−21	
				392.3	1	−20	−24	−26	

* quantitative ions.

**Table 2:** Parameters of the method for quantification of prostaglandins in pharmaceutical drugs.

Calibration Curve			Validation Results				
Substance	Internal Standard	The Coefficient of Determination (*R*^2^)	Concentration Level [µg/mL]	Intraday		Interday	
				Precision [%] *	Accuracy [%] *	Precision [%] *	Accuracy [%] *
Carboprost	Dinoprost-*d_4_* (PG-F2α-*d_4_*)	>0.9988	0.1 1 10	2.9 2.4 3.3	−4.5 −3.8 1.4	3.0 4.4 1.2	0.3 −0.2 −0.6
Cloprostenol	Dinoprost-*d_4_* (PG-F2α-*d_4_*)	>0.9988	0.1 1 10	2.4 1.5 2.4	4.3 −4.1 1.8	2.5 1.0 1.1	4.0 −3.8 1.0
Dinoprost (PG-F2α)	Dinoprost-*d_4_* (PG-F2α-*d_4_*)	>0.9980	0.1 1 10	5.0 4.2 1.4	3.0 −0.9 3.6	3.8 4.7 2.4	5.0 2.6 3.0
Dinoprostone (PG-E2)	Dinoprost-*d_4_* (PG-F2α-*d_4_*)	>0.9977	0.1 1 10	4.4 3.5 1.8	4.3 −3.1 4.3	2.4 0.8 0.7	4.7 −0.9 3.0
Misoprostol	Misoprostol-*d_5_*	>0.9991	0.1 1 10	1.0 4.2 2.2	5.0 2.1 4.8	4.2 2.2 1.6	4.0 −1.2 3.1
Sulprostone	Dinoprost-*d_4_* (PG-F2α-*d_4_*)	>0.9988	0.1 1 10	1.9 4.1 4.9	5.0 2.7 −3.4	3.5 4.5 2.9	4.0 2.0 −1.4

* (n = 3).

**Table 3:** Summarization of methods applied for prostaglandins analysis in pharmaceutical drugs.

Method	Prostaglandins	Details of the Method	Tested Samples	Qualitative/ Quantitative	Year	Reference
ATR-FTIR	Misoprostol	Characteristic wavelengths: 3304, 1737, 1364, 1016 cm^−1^	Pills	Qualitative	2022	[14]
Spectrophotometry	Misoprostol	Absorbance measured at 247.6 nm	Pills	Quantitative	2011	[15]
HPLC-UV	Carboprost	Chromatographic column: Symmetry C18 (100 × 4.6 mm × 3.5 µm); Absorbance measured at 200 nm;	Injection liquids	Quantitative	2014	[16]
LC-MS	Misoprostol	Comparison of chromatograms obtained during analysis of authentic samples with chromatograms obtained for Cytotec^®^ tablets (reference material)	Abortion pills collected from vagina during gynecological examination	Qualitative	2019	[24]
LC-QTOF-MS/MS	Carboprost Misoprostol	Chromatographic column: Suplex™PKB (250 × 2.1 mm × 5 µm); Mass spectrometry: ESI (sodium adducts in positive ionization); MRM	Infusion preparations	Quantitative	2007	[18]
LC-QTRAP-MS/MS	Misoprostol	Chromatographic column: Capcell PAK C18 MGII (50 × 2.0 mm × 3 µm); Mass spectrometry: ESI (negative ionization); MRM	Counterfeit drugs distributed in the illegal marketplaces	Quantitative	2019	[17]
UHPLC-QqQ-MS/MS	Carboprost Cloprostenol Dinoprost (PG-F2α) Dinoprstone (PG-E2) Misoprostol Sulprostone	Chromatographic column: Acquity UPLC BEH C18 (50 × 2.1 mm × 1.7 µm); Mass spectrometry: ESI (sodium adducts in positive ionization & negative ionization); MRM	Abortion pills collected from vagina during gynecological examination	Quantitative	2022	Presented method
